# What Do Mothers (or Caregivers) Know about Their Children’s Oral Hygiene? An Update of the Current Evidence

**DOI:** 10.3390/children9081215

**Published:** 2022-08-12

**Authors:** Riccardo Aiuto, Mario Dioguardi, Silvia Caruso, Erica Lipani, Dino Re, Roberto Gatto, Daniele Garcovich

**Affiliations:** 1 Department of Biomedical, Surgical and Dental Science, University of Milan, 20122 Milan, Italy; 2 Department of Life, Health and Environmental Science, University of L’Aquila, 67100 L’Aquila, Italy; 3 Department of Clinical and Experimental Medicine, University of Foggia, 71122 Foggia, Italy; 4 Department of Surgical and Dental Sciences, University of Cagliari, 09121 Cagliari, Italy; 5 Department of Dentistry, Universidad Europea de Valencia, 46010 Valencia, Spain

**Keywords:** parents, caregiver, children, tooth decay, children’s oral hygiene, prevention

## Abstract

Tooth decay remains one of the most common diseases in children, although it is a preventable injury and despite significant advances that has been made in terms of attention and care for oral hygiene. Several studies have shown the association between prevalence of tooth decay in children and parents’ incorrect oral care habits, with a low educational level and a low socioeconomic background. The question that arises concerns the actual oral hygiene knowledge of mothers, fathers, family members or caregivers of young patients; therefore, the aim of this review is to investigate the genesis of gaps in the topic. A literature search was conducted through the Scopus and PubMed search engine and ended in May 2022; only studies from the past 20 years were included. Current evidence suggests that parents and caregivers still have little knowledge about their children’s oral health: there is not enough awareness about the importance of preventing oral diseases, due to poor attention to good oral hygiene but also lack of information from health professionals and institutions. In the future, all the professionals involved in the pregnant woman’s care should increase parents’ knowledge, solve their doubts, collect and compare data in order to design effective intervention programs.

## 1. Introduction

Despite considerable progress in terms of attention and care for oral hygiene, already observed in the previous century, tooth decay remains one of the diseases with a high prevalence globally [1]. A global study published by The Lancet in 2017 estimates that 2.3 billion people suffer from tooth decay in permanent teeth and more than 530 million children suffer from decay of deciduous teeth [2].

A more recent Italian research shows the prevalence of Early Childhood Caries (ECC), which is close to 15% in the age group between 4 and 6 years in a sample of 3000 children; the even more significant result, however, is represented by the association between caries prevalence and parents incorrect oral care behaviors [3]. As further evidence of this, parents often take children under the age of 6 months to the dentist for dental pain, when the carious lesion is already in an advanced stage [4]. Many parents are not fully aware of the strong association between processed sugar-rich food and beverage consumption and the onset of EEC [5].

The World Health Organization (WHO) established a series of oral health goals to be achieved by 2020; among these, one of the most ambitious was to obtain 95% caries-free subjects in the 5 to 6 years cohort [6]. The goal, based on a too optimistic forecast, did not coincide with the current reality as highlighted by recent cross-sectional studies [7]. The multiple efforts made in the last decade resulted in a general improvement, but it must be taken into account that preventive strategies, such as water fluoridation, could not be successful if awareness of correct oral hygiene habits is not improved at a global level.

The objective of this review is to investigate the genesis of gaps in oral hygiene knowledge of mothers, fathers, family members, or caregivers of young patients. Awareness of this topic can indicate to the pediatric dentist and other health professionals, at what level, first, to educate and, second, to intervene to improve prevention in either a public or private health-care system. Among other health professionals, the general dentist may play a crucial role; moreover, it is this professional who takes care of the family’s dental health and is often the one with whom the child has his/her first dental visit.

Despite its preventable nature, caries is still one of the most prevalent diseases in children [8], five times as common as asthma and seven times as common as hay fever [9]. The impact of Early Childhood Caries (ECC) is disproportionately higher in subjects with a low socioeconomic background [10]. In the US, the prevalence of ECC in children of low socioeconomic status, with parents with a low educational level and a high-sugar diet, is 32% higher than in the general population [11]. Unfortunately, frequent and early exposure to sugary foods is common and inevitably influences future food preferences and behaviors [5]. Establishing an appropriate oral hygiene protocol at an early stage is essential to effectively reduce the impact of this silent epidemic.

The prevalence of caries in a large cohort of Spanish individuals aged 5 to 6 years showed a significant increase in the last two decades, with the worsening especially marked in recent years [12]. This trend, despite the existence of community oral health programs, indicates that community dental care programs, particularly preventative activities, should be reinforced starting in the first year of life, with a particular attention to the most disadvantaged groups. Dental public health should promote and ensure community-based education in various venues, such as schools, community centers, medical clinics, and support initiatives such as “first tooth first dental visit”, especially in districts with low socioeconomic conditions.

Periodontal problems are also of great concern in adolescent age; however, the strategies suggested to be implemented to prevent their onset are similar: prevention programs and self- assessed oral health [13].

Preventive protocol and procedure and ECC are among the topics of greatest interest in pediatric dentistry research [14]. From a first survey of the current literature, it was clear that the studies that investigated the knowledge of oral hygiene in pediatric dentistry were very heterogeneous, as they adopt very different protocols and samples. Therefore, the data could not be aggregated and the authors decided to complete a narrative review of the subject. The literature search was conducted through the Scopus and PubMed search engine and ended in May 2022. The combination of the following keywords was used to search for the articles: awareness, knowledge, beliefs, oral health literacy, perception (“What do”); mothers, fathers, caregivers, parents, guardians, pregnant (“mothers/caregivers know about”); child, newborn, infant, children, toddler (“their children’s”); oral hygiene, prevention (“oral hygiene”); community health providers and dental public health (“dental public health”). Only studies from the past 20 years on the subject were included.

## 2. Parental Influence on Children’s Oral Health Practice and Status

From a historical point of view, the researchers first investigated the biological and dietary determinants of the oral health status of children, but more recently a more complex framework has been proposed to explore the effects on children’s oral health. The proposed framework included biological and genetic determinants as well as those from the social and physical environment and those related to health influencing behaviors and medical care [15]. Several studies have also suggested the possibility that dental caries could be correlated with anxiety and stressful situations; in fact, a positive correlation was found between salivary cortisol levels and the experience of tooth decay in children [16]. Several connections are observed between maternal salivary bacterial challenge and children’s dental health: countless studies report that vertical transmission, especially of Streptococcus mutans, from mother to child is strongly associated with an increased incidence of ECC [17,18,19]. Therefore, ECC prevention strategies should include measures to prevent or delay early colonization by cariogenic bacteria [20].

The oral health status of children can be influenced by many different factors related to their oral health practices, but also to parental factors. Children, up to age 5, spend most of their time at home with their primary caregivers, even if attending kindergartens or nurseries. During this period, “primary socialization” occurs and children begin to acquire daily living skills and knowledge from their parents and caregivers. Dietary and oral hygiene practices are among the living skills that children acquire from their parents. Therefore, there is a correlation between the oral health status of children and the knowledge and behaviors of parents, caregivers, and older siblings [21]. Various variables, including attitudes, contribute to specific behaviors. If behaviors are learned and established before age 5 years, they are likely to become deeply established. Therefore, early childhood constitutes an important period for future oral health [22]. As previously highlighted, factors such as family educational level and gross income are negatively related to caries incidence [23]. According to Broadbent et al. [24], socioeconomic status, as well as beliefs about oral health care (held by individuals and their parents) in early life, are related to oral health-related behaviors in early adulthood [24]. Salama et al. [25] remarked the strong link between educational level and oral health literacy, but also highlighted how, despite social status, most of the caregivers are still not aware of the importance of prevention. According to their results, 46.7% of children up to 5 years of age never visited the dentist, 41.8% had bottle feeding on demand, and only between 8 and 22% of the children, depending on their family income, had their first dental examination [25]. Therefore, in this area, appropriate advice on children’s oral care is not easily obtained from dental professionals.

Hiratsuka [26] highlighted in a sample of Alaskan residents that, although most parents did not achieve the appropriate frequency of oral hygiene, the odds ratio of children who received the required amount of toothbrushing was 49 times higher if their parents are used to brushing their teeth a minimum of twice a day [26]. In a Mexican sample Vallejos-Sanchez et al. highlighted that a greater mother’s approach to oral health and dental care was associated with a 2.4 higher chance of frequent toothbrushing in the child [27]. Finlayson et al. reported that brushing frequency in African American children was strongly associated with mother’s brushing behavior [28].

Once the current evidence is reviewed, the role of oral health education programs becomes controversial. Improvements in parents’ knowledge are generally associated with short-term improvement in children’s plaque index and gingival index [29]. Very few studies were conducted to assess the incidence of dental caries after health education. According to the results of Frazao et al. in 2011, a school-based supervised toothbrushing program could be effective in preventing caries [30]. Children showed a significant improvement in toothbrushing skills when demonstration and supervision were provided.

There is indeed a need to understand children’s and parents’ knowledge to develop more effective approaches to oral health promotion. Periodical reinforcement of educational programs is fundamental in order to obtain a significant result in the long term. Shaghaghian et al. in 2016 [31] presented a study of 396 parents and their 3 to 6 years old children, where parental awareness was determined by comparing parents’ perception of their child’s oral hygiene with the results of the dental examination. The findings demonstrated that 40.9% of parents (169) were unaware of their child’s dental cleaning status. Awareness was strongly related to the oral hygiene of the children and the parental level of employment and education, as well as socioeconomic status. The results of the study reinforce once again the need for educational interventions especially aimed at young families with low socioeconomic status and parents of children with poor oral hygiene [31]. This intervention could be effective in increasing parental knowledge and skills that help them recognize their child’s dental needs.

The socioeconomic status of parents is not an indifferent variable; Sweden, Denmark, and Norway, through Public Dental Services (PDS), provide free dental care, thus considering the needs of socioeconomically vulnerable individuals and groups [32]. The “Nordic model” is a successful system that has allowed, on the one hand, reduction in the incidence of tooth decay in children and adolescents, and on the other hand, collection and reporting of caries data at a national level [33].

As an example, Greenland, through a national caries strategy (CSG), was able to significantly improve caries status without increasing costs. The program provided a first dental approach at the age of 8 months, followed by visits based on nine selected dental ages during which dental health education and promotion took place before the develop of caries; fluoride products and dental sealants were essential [34].

In a study that included parents of five-year-old Irish children, it was highlighted how parents generally display limited knowledge about the dental and oral health of their young children. The authors indicate the need to improve parents’ education, especially about toothbrushing behavior and the use of fluoridated toothpaste [35]. Daly et al. followed for three years 1345 parents from year 1 to year 4 of their child development and highlighted how parents’ perceptions of the care of their children’s teeth and/or gums improved over time [36]. Parents consistently perceived that they provided better medical care than dental care for their child, probably because there is still a tendency to consider dental health as not as critical as general health care [36].

According to the data presented by Aiuto et al. on 763 pregnant women in the Italian population, young women too often tend to underestimate the importance of oral care, even before pregnancy, and this attitude persists throughout gestation and even after the birth of the child [37]. Dentist examinations are often limited to urgent treatments, and the awareness of preventative strategies is poor. This type of oral health approach can result in the development of pathologies such as caries or periodontitis in both children and caregivers. Furthermore, many parents do not consider the multi-disciplinary nature that a dental exam could offer; suffice it to say that children diagnosed with occlusal defects should be evaluated and may receive an early diagnosis of visual defects [38]. Pregnant women should receive adequate education through doctors, healthcare providers, social workers, and childbirth courses [37]. In other population groups also, as reported by Gaffar et al. in an Arabic sample, only a few pregnant women seek regular dental counseling before and after pregnancy [39].

Gaszyńska et al. in 2015 after comparing perceived oral health with the actual oral health status of 1345 pregnant women concluded that the compatibility of the subjective and objective oral health evaluations was very low [40]. Most of the pregnant women had an overly optimistic judgment about their oral health status. Despite that 78.1% recognized the importance of maintaining good oral health during pregnancy for the baby’s general wellbeing, only 1 in 2 women (53%) visited a dentist during pregnancy. Despite the strict medical check-up schedule provided by the welfare system, there was a negligible improvement in awareness of the negative effects of poor oral health of a mother on the fetus and neonate [40]. A cross-sectional survey involving 947 German midwives highlighted how only 53.5% of midwives recommended a dental visit during pregnancy, and though 60.4% recommended that oral hygiene begins with the eruption of the first tooth, only 26.8% offered advice about the frequency of tooth brushing and 1.3% about the dosage of toothpaste. The results of the investigation underlined the variability of the information given to pregnant women by one of the main sources of information during pregnancy [41,42].

Parents should be also informed on oral device safety: in fact, it has been reported that the use of orthodontic pacifiers instead of the classic ones did not promote the onset of pernicious oral habits in children in primary dentition, despite their prolonged use [43].

In a pediatric context, there is a substantial evidence in the medical literature that indicates significant associations between caregiver generic health literacy and children’s general health status. In pediatric dentistry, the relationship between caregiver OHL and children’s oral health status is not yet clear, although it is acknowledged that caregivers play a critical role in the prevention and management of children’s oral health status. A study in the Asian population by Bridges et al. reported a strong association between OHL caregivers and children’s oral health status [44]. However, other studies reflect how the level of oral health literacy does not always guarantee a good oral health status, as highlighted in a study of Latino caregivers in Colorado, with children up to six years of age. Caregivers have some understanding of the factors associated with dental caries and knowledge about oral hygiene guidelines, but their knowledge did not translate into positive oral health behaviors. Parents discussed their child’s resistance to brushing teeth and the lack of time to supervise the children during brushing as some reasons for poor oral hygiene for their children. The authors suggest how culturally tailored oral health education is required for this population [45].

A multicentric study enrolling 2822 parents and children aged 3 to 4 years of age from a wide range of countries, and diverse ethnic groups, from deprived and nondeprived backgrounds and for those with children who had experienced caries and those who had not, clearly demonstrated that parental attitudes towards oral health related behaviors do influence whether these behaviors are reported to occur in children. The study found significant differences in favorable health-related attitudes in parents of children with and without caries, irrespective of material deprivation [46]. For all the reasons mentioned above, it is of paramount importance to provide counseling and advice from a very early stage to caregivers and children. The information should be consistent to avoid confusion and a consensus should be reached among all the professionals involved, dentists, gynecologists, pediatricians, and midwives, among others. The information and education action plan should include long-term actions and interventions to improve its efficacy.

## 3. Home Oral Care of Children

### 3.1. Recommended Standards of Oral Hygiene 0 to 6 Years of Age

The American Academy of Pediatric Dentistry (AAPD) and the European Academy of Paediatric Dentistry (EAPD) do agree that oral hygiene measures should be implemented no later than the eruption of the first primary tooth. Parents should perform or assist with brushing in preschool children using a toothbrush adapted to the child’s age, with a fluoride toothpaste amount that varies according to the child’s age [47].

The AAPD recommends, in children under the age of three, a smear or rice-sized amount of fluoridated toothpaste, while in children ages three to six, a pea-sized amount of fluoridated toothpaste [48]. The EAPD recommends the same amount of toothpaste in preschoolers but with 1000 ppm of fluoride [49]. Both authorities agree that after brushing, rinsing with water should be kept to a minimum or avoided to maximize the protective effect of fluoride.

According to Marinho in 2009 the use of fluoridated toothpaste has the highest caries preventive effect when compared to brushing frequency, supervised toothbrushing and fluoride concentration, highlighting the importance of implementing this procedure on a daily basis [50]. EAPD recommends, also in preschool children, brushing for more than one minute, reaching every tooth surface.

### 3.2. Compliance with Fluoride Recommendation

According to the values reported in Table 1, the use of fluoride toothpaste displays a huge variability among different countries and population subgroups. The most striking result is that in many of the included studies, a large part of the sample was unaware of the kind of toothpaste used. Therefore, it is arguable that, if a patient is unaware of the presence of fluoride in his toothpaste, he is probably unaware of the optimal dose according to age. Several authors agree that the widespread use of fluoride toothpaste is one of the key factors in the decrease in carious lesions in the specific society [51].

Alvey in a study on 119 first-time English-speaking pregnant women interviewed before and 12 months after birth highlighted how knowledge regarding fluoride exposure between the cohorts concerning fluoride exposure displayed a significant percentage of mothers who have not implemented this prescribed practice. About one-third of the participants neither intended to implement a fluoridated toothpaste nor ever used fluoridated toothpaste [58]. According to Nguyen in 2017, parents show mixed and limited knowledge and beliefs about the role of fluoride in caries prevention despite the large body of evidence on the effect of fluoride [56].

In Germany, for example, there is a lack of consensus among pediatricians and dentists regarding proper use and the amount of fluoride that should be contained in toothpastes for children 0 to 3 years old. The German Society of Oral and Maxillofacial Surgery, in agreement with the AAPD and the EAPD, recommends that baby’s tooth brushing using a fluoride-enriched toothpaste should start when the first tooth emerges. If a child is able to spit toothpaste, the German Society of Paediatrics and Adolescent Medicine and the German Academy for Paediatrics and Adolescent Medicine suggest that he or she begin using it by the age of 4. Persistent failure to agree on the preventive procedures in the first 3 years of life could lead to parental uncertainty about the benefit of fluoride [41].

According to what Prabhu et al. 2013 highlighted, most of the parents involved in the sample (76%), despite the regular use of toothpaste, are unaware of which kind of fluoride toothpaste they are using to brush their children’s teeth [21]. The results reported by Gussy et al. in 2008 [59] report that in his study group of 308 Australian caregivers there was confusion about whether a fluoride paste should be used at all with toddlers. The majority of parents (55%) did not know whether fluoridated toothpastes should be used with toddlers, 31% believed they should not and 13% believed they should be used [59]. According to what was recently reported in a Brazilian cohort, educational programs and interventions had positive effects on children’s oral health-related behaviors and caregivers’ oral health knowledge, improving fluoride toothpaste use in the right amount according to the age of the intervention group [60].

### 3.3. Compliance with the Oral Hygiene Recommendation

In many countries early preventive dental care is generally not performed in preschool children; current literature reports that this is the result of several factors, including: lack of knowledge and awareness of the importance of primary teeth, parents’ dental fear and popular misconceptions about dental care [61]. Roberts & Codon reported how participants in a UK survey believe that oral health care is simple, common sense and something everyone knew, without considering the importance of being properly educated in this field [62]. Although it is the desire and interest of parents that their child is at his or her best and their teeth are healthy, the result of this mindset is they do not seek further preventive care information or advice, and the information offered to parents is frequently considered opportunistic [62].

As reported in Table 2 in most of the available studies, the frequency of brushing is still suboptimal even in European countries. Although the number of people who brush less than once a day is significant but low, in most countries the majority of the population brush only once a day at preschool age. It is therefore clear how the awareness of toothbrushing guidelines is low or, at least, they are not properly enforced.

### 3.4. Assisted Toothbrushing

As clearly stated in the guidelines of both the AAPD and the EAPD, the role of caregivers is to assist in and perform good oral hygiene practices their children under the age of six years.

According to the result of the evidence resumed in Table 3, the percentage of caregivers who do not assist their children while brushing or that supervise them on a non-daily basis is very high. As reported by Huebner et al. 2010 [73], not assisting children’s oral hygiene can not only reflect a lack of guidelines knowledge, but be the result of a series of personal barriers or external constraints. Personal barriers are the wrong oral health beliefs (tooth brushing is harmful to enamel), children’s emotional reactions, low caregivers’ self-standard of care, or lack of manual skills. On the other hand, external constraints are related to busy modern life and tight daily schedules. It is worth underlining that, in the Huebner study population, almost all (91%) parents considered that brushing child’s teeth twice a day was reasonable. However, in practice little more than half (55%) claimed to have reached the goal [73].

Scientific data based on self-report have to be handled with care; the results may, in fact, be an overestimate due to social desirability response bias, h is a common problem to take into account in this type of study.

### 3.5. Brushing Start Age

As reported in Table 4, it can be noticed that parents are generally not aware of the correct age to start brushing their child’s teeth. A survey in a sample of North American parents revealed that only a small percentage of parents (25.7%) actually know the starting age when a child should start receiving healthcare, and again a low percentage of correct answers were recorded regarding when a mother should start brushing a child’s teeth (32.4%). The authors speculated that these percentages may be the result of the low consideration that mothers may place on taking care of children’s primary teeth [76].

Nuñez Correia et al. in 2017, exploring the plans that mothers-to-be had about their children’s dental care, underlined that 26.1% of the participants did not think about when to begin cleaning their children’s teeth, while 7.8% were uncertain about the timing [81]. Almost half of the mothers interviewed correctly intended to start brushing their baby’s teeth as soon as the first tooth emerged, but 20% would first seek the counsel of a health professional, 13.9% were unsure, and 11.3% planned to do this when the baby started eating solids. Their findings highlighted that currently in the UK half of pregnant women are unaware of the correct brushing timing [81].

### 3.6. Family Behavior Modification

Oral health education is fundamental in preventing dental caries in children, with the goal of changing the knowledge, attitudes, and behaviors of the patient and the caregiver of the patient that put children at risk of oral illness; however, the child’s ability to assimilate and mimic the good as well as bad habits of adults cannot be ignored [8].

As outlined by Naidu et al. in 2020, also in settings characterized by positive attitudes toward preventive oral healthcare, a variable degree of uncertainty regarding dental attendance, supervised brushing, fluoride use, and sugar intake could be outlined. These findings suggest that these items require a special emphasis in oral health promotion programs targeted at improving early childhood oral health [82].

As outlined by the WHO, oral health education and community engagement are essential for preventing early childhood caries especially in a low to middle-income countries. The family represents the child’s primary source of learning about oral health and risk factors. The WHO suggests improvement in the awareness of oral health and ECC prevention among parents through proper health communication and the provision of sound information on disease prevention and treatment [83].

Evidence suggests the effectiveness of behavioral therapies against ECC when used at the individual and family levels [84]. The motivational interview is a promising approach to induce positive changes in caregivers’ oral health knowledge and child behavior outcomes. The results are improved when delivered in a patient-centered environment, rather than in the potentially stressful and distracting environment of a busy dental clinic [85].

In a recent report, Toniazzo et al., 2019, outlined how mobile applications and text messages can be a promising means to increase oral health awareness, of young children’s parents, and promote stable behavioral change [86].

A single, low-cost, low-intensity intervention could significantly reduce the risk of new caries: Pine et al., 2020, in fact, have shown how efficient dental nurse intervention could be, based on motivational interviewing and focused on prevention of future caries in children who have undergone primary tooth extraction. [87].

## 4. Conclusions

Parents and caregivers still know too little about their children’s oral health: in general, caregivers are not sufficiently aware of the importance of preventing oral diseases, due to poor attitudes to good oral hygiene and lack of information by health-care professionals and institutions. Information gathered in an informal way, often on the Web, can be misleading. A correct flow of evidence-based information should be established involving health care professionals at different levels. Dentists, pediatric dentists and all those involved in the care of a pregnant woman, such as medical doctors, midwives, health workers or social workers, should help to increase the awareness of parents and caregivers of oral health and solve the doubts that can arise in the perinatal period and in the first years of a child’s life.

The different stakeholders should establish by consensus a validated protocol to assess patients’ literacy on aspects such as prevention and oral health. It will thus be possible to collect and compare a greater amount of data, coming from different social and ethnic backgrounds, and highlight the current weakness and strengths. Such evaluation could help to design effective action programs that address more specific targets, both in private and public national health systems.

## Figures and Tables

**Table 1:** Frequency of use of fluoridated toothpaste among different populations. Author, country and sample size (N) are reported. Low fluoride < 1000 ppm–Standard fluoride ≥ 1000 ppm.

							Fluoride Toothpaste		
	Country	N	Age	Do not Know/No Answer	No Toothpaste	Non Fluoridated	Fluoridated Toothpaste	Low Fluoride	Standard Fluoride
Buckeridge A. et al., 2020 [52]	Australia	155	2–6		13%	29%		50%	8%
Hobbs et al., 2020 [53]	New Zealand	4723	0–14	4.70%	1%	6.40%		8%	79.90%
Avenetti D. et al., 2020 [54]	USA	148	1–2	35.9%	3.7%	13.7%	50.4%		
Kumar G. et al., 2019 [55]	India	500	N/S		1.20%	4.00%	94.8%		
Nguyen U.N. et al., 2017 [56]	USA-Vietnamese	45	1–5	27%		11%	59.0%		
Guerra F. et al., 2017 [57]	Italy	103	3–17	36.4%		22.7%	40.9%		
Prabhu A et al., 2013 [21]	India	150	0.7–4	36.7%		42.7%	20.7%		

**Table 2:** The daily frequency of toothbrushing reported in recent literature.

				Frequency of Tooth Brushing		
Authors/Year	Country	N	Age	<1	1	≥2
Buckeridge A. et al., 2020 [52]	Australia	155	2–6	6.0%	46.0%	48.0%
Zhang Y. et al., 2020 [63]	Hong Kong	455	5–7		26.4% †	73.6%
Hobbs et al., 2020 [53]	New Zealand	4723	0–14	4.70%	26%	64.90%
Avenetti D. et al., 2020 [54]	USA	148	1–2	14.2%	26.4%	59.5%
Alshammary F. et al., 2019 [64]	Middle-east	233	2–6	6.2%	22.9%	70.9%
Kumar G. et al., 2019 [55]	India	500 §		0.80%	10.40%	88.80%
Pan N. et al., 2017 [65]	China	1042	9	1.6%	13.4%	84.9%
Pan N. et al., 2017 [65]	China	1042	11	0.8%	19.6%	79.9%
Encuesta de salud Oral 2017 [66]	Spain	N/S §	3–4	13.0%	34.4%	52.6%
Nguyen U.N. et al., 2017 [56]	USA -Vietnamese	45	1–5	30%%	9%%	61%%
Guerra F. et al., 2017 [57]	Italy	103 §	3–17	1.0%	85.4%	13.6%
Alyahya L. 2016 [67]	Kuwait	236	N/S	10.1%	38.1%	51.7%
Vermaire & Van Exel 2018 [68]	Netherlands	170 §	6–9	N/S	N/S	43.6%
El Karmi et al., 2015 [35]	Ireland	114	5	N/S	N/S	80.70%
NHANES 2014 [69]	USA	449	3–5	0.3%	37.5%	62.2%
Prabhu A. et al., 2013 [21]	India	150	0.7–4		76.0%	24.0%
Vermaire J.H. et al., 2010 [70]	Netherlands	290 §	6	21.3%	33.1%	45.5%
Chhabra & Chhabra 2012 [61]	India	653	1–4	46.90%	41.30%	11.80%
Leroy R. et al., 2011 [71]	Belgium	974	3	29.0%	54.0%	17.0%
Leroy R. et al., 2011 [71]	Belgium	998	5	20.0%	57.0%	23.0%
Castro Martins C. et al., 2011 [72]	Brazil	197	3–4	14.1%	49.5%	36.4%
Chu C.H. et al., 1999 [23]	Hong Kong	658	4–6	13.0%	44.0%	43.0%

N/S Not Specified within the text; § The type or caregiver is not reported within the text; † Includes all the individuals who started after the age of 12 months.

**Table 3:** Prevalence of assisted tooth brushing.

				Assisted Tooth Brushing		
	Country	N	Age	Yes	No	Not Daily
Avenetti D. et al., 2020 [54]	USA	148	1–2	65.70%	0.70%	33.60%
Nguyen U.N. et al., 2017 [56]	USA-Vietnamese	45	1–4	0%	25%	75%
Vermaire & Van Exel 2018 [68]	Netherlands	170	6–9	33.30%	N/S	
Alyahya L. 2016 [67]	Kuwait	236	N/S	61.90%	38.10%	
El Karmi et al., 2015 [35]	Ireland	114	5	21.90%	78.10%	
Chhabra & Chhabra 2012 [61]	India	653	1–4	36.50%	63.50%	
Vermaire J.H. et al., 2010 [70]	Netherlands	290§	6	70.70%	2.40%	26.90%
Castro Martins C. et al., 2011 [72]	Brazil	197	3–4	65.00%	35.00%	
Leroy R. et al., 2011 [71]	Belgium	974	3	51%	6%	43%
González Martínez F. et al., 2011 [74]	Colombia	333	1–5	68.40%	10.10%	21.50%
Chan S.C.L. et al., 2002 [75]	Hong Kong	260	1–3	44.00%	56.00%	
Chu C.H. et al., 1999 [23]	Hong Kong	658	4–6	56%	44%	

N/S Not Specified within the text.

**Table 4:** Age at start of brushing in different population groups.

						Age at Start of Brushing			
	Country	N	Age	Don’t Know	Not Yet Started	≤1	> 1 < 2	≥2	≥3
Buckeridge A. et al., 2020 [52]	Australia	155	2–6		5%	68%	27% †		
Leroy R. et al., 2011 [71]	Belgium	974	3		1%	34%	44%	17%	
Leroy R. et al., 2011 [71]	Belgium	998	5		0	25%	32%	40%	
Calcagnile F. et al., 2019 [77]	Italy	101	3–5		1%	30% ‡		57%	12%
Vozza et al., 2017 [78]	Italy	304	0–3		53.1%	22.5%	23.7%		
Rigo L. et al., 2016 [79]	Brazil	79	1–3	16.5%		72.2% ‡			20.30%
Hoeft et al., 2009 [80]	USA	82	5–10			13%	41%	45%	

† Includes all the individuals who started after the age of 12 months. ‡ The questionnaire asked if tooth brushing started after the eruption of the first tooth; we assumed the first tooth to erupt around the age of 4 months.

## Data Availability

The data that support the findings of this study are available from the corresponding authors [R.A. and E.L.], upon reasonable request.
